# Health Equity in All Urban Policies: A Case Study of Richmond, California

**DOI:** 10.1177/2752535X241273955

**Published:** 2024-08-13

**Authors:** Jason Corburn, Shasa Curl, Gabino Arredondo

**Affiliations:** 1 Deaprtment of Public Health, 1438 University of California Berkeley, Berkeley, CA, USA; 2 94039 City of Richmond, Richmond, CA, USA

**Keywords:** urban health equity, governance, health in all policies built environment

## Abstract

Local governments working in partnership with communities can institutionalize practices that promote health equity. We offer a case study of how one city in the US is implementing Health in All Policies (HiAP) with the explicit aim of promoting health equity. We use participant observations, original document reviews and interviews to describe how Richmond, California, is building new partnerships, programs and practices with community-based organizations and within government itself as part of the implementation of its HiAP Ordinance. We also report on indicators that were identified by community and government stakeholders for tracking progress toward improving place-based determinants of population health. We find that the responsibility for implementing Richmond’s HiAP Ordinance rests on a new institution within local government and this entity is building new partnerships, promoting innovative policies and augmenting practices toward greater health equity. We also reveal how city governments and community partners can collaboratively track progress toward health equity using locally gathered data.

## Introduction

City government are increasingly working with community partners to implement laws and practices that can address the drivers of systemic heath inequities, including but not limited to poverty, spatial discrimination, and structural racism, and promote greater health equity.^1^ This case study explores how one working class, majority Black, Indigenous and People of Color (BIPOC) city in the San Francisco Bay Area, called Richmond, California, is using a Health in All Policies (HiAP) approach to pursue health equity. HiAP recognizes that health is created by a multitude of factors beyond healthcare and, in many cases, beyond the scope of traditional public health activities.^2^ WHO’s Helsinki Statement on Health in All Policies described HiAP as “an approach to public policies across sectors that systematically takes into account the health implications of decisions, seeks synergies, and avoids harmful health impacts in order to improve population health and health equity.”^3^ The US Centers for Disease Control and Prevention defines HiAP as “a collaborative approach that integrates and articulates health considerations into policymaking across sectors to improve the health of all communities and people.” The National Association of City and County Health Officials defines HiAP as “a change in systems that determine how decisions are made and implemented by local, state, and federal governments to ensure that policy decisions have a neutral or beneficial impact on health determinants.”^4^

Community health practitioners have recognized that HiAP implemented at the local level may best promote equity since this is the scale closest to decisions that influence everyday opportunities to make healthy choices and avoid unhealthy exposures.^5–7^ Yet, very little is understood about what specific practices and partnerships might constitute HiAP implementation within city government and how communities and cities are tracking progress toward local health equity. This case study describes the programs, policies and practices that fall under Richmond, California’s HiAP Ordinance, how they are being implemented, and how the city and community partners are tracking progress toward health equity.

The City of Richmond, California is a post-industrial, largely immigrant and African American city of about 120,000 people in the San Francisco Bay Area. Richmond is the former industrial home of the Bay Area, with a large port and petrochemical refinery, among other industries. Richmond also has a long history of Black organizing. For example, during WWII shipyard workers organized to demand fair wages and access to quality public housing. In the 1960s Richmond organizers helped launch the Black Panther Party and by the 1980s organizers formed one of the first urban environmental justice organizations in the US called the West County Toxics Coalition. In the 1980s and ‘90s, the city experienced massive industrial decline, unemployment, an influx of refugees from war torn Central America and Southeast Asia, a crack epidemic and a military-style response to crime including mass incarceration of young Black and Brown male residents. By the early 2000’s Richmond was the 9^th^ most violent city in America (measured by gun homicides), had a life expectancy of 73 years compared to the regional mean of close to 80 years, and residents in the city had the highest prevalence of chronic illnesses of any city in the SF Bay Area.^8,9^

As one response to these health inequities, Richmond began a series of strategies with community partners to promote greater health equity. Richmond does not have its own department of public health and must rely on its county Health Services Department that services over 1.2 million people across 717 square miles. One locally-specific population health strategy occurred in 2005 when Richmond became the first city in California to draft and adopt a Community Health and Wellness Element or chapter into its General Plan. The *Health Element*, as it was called, was co-created by tens of community-based organizations and hundreds of residents, who collaborated to set equity-oriented planning and policy goals.^10^ At the request of community groups who did not just want a plan but tangible actions, the City established the Richmond Health Equity Partnership (RHEP) in 2010 as a way to implement recommendations from the Health Element. The RHEP was made up of CBOs, the school district, county and city government officials and its three charges were to implement actions identified by the Health Element, create a health equity ‘score card’ and to further institutionalize health equity within Richmond’s government. The RHEP helped implement projects such as park improvements, roadway and intersection safety measures, school-based feeding programs, neighborhood clean-ups and youth employment and training.^10^ The score-card process identified indicators of health equity to track over time.^10^ The RHEP also recommended that the City draft and adopt a Health in All Policies Strategy in-order to further institutionalize its commitment to health equity.

A sub-group of community-based organizations, representatives from government agencies and academic institutions worked collaboratively for 18 months to draft Richmond’s HiAP Strategy.^11^ The Strategy formed the basis of Richmond’s HiAP Ordinance, which was unanimously approved by the Richmond City Council in 2014. By adopting a HiAP law, Richmond, California became the first city we know of in the US to legislate the use of HiAP. As we explain further below, Richmond’s HiAP Ordinance was intended to act as a guiding framework for new, health-equity focused community-based partnerships, to generate new, interagency practices and to establish a transparent indicator tracking system to measure place and population-based progress toward greater health equity. This paper presents a descriptive case study of the implementation practices of Richmond’s HiAP Ordinance, community and practitioner perceptions of whether or not these practices are promoting health equity and the indicators selected to measure progress toward change. Previously published material has detailed the processes behind the drafting and overall health equity approach of Richmond’s HiAP.^10,12,13^

## Methods

We used a case study approach to present the implementation practices within Richmond HiAP Ordinance. This case study uses observation of practice, interviews, and secondary data to ‘triangulate’ evidence and offer an understanding of a complex practice such as HiAP.^14^

### Data Collection

To construct the case study, we first reviewed all internal meeting notes and minutes from 52 community and city meetings held between 2014 and 2019, about Richmond’s HiAP implementation. These were meetings organized by the City of Richmond’s Interdepartmental HiAP Team (described below). Using these texts, we identified projects, partnerships and programs that were linked to HiAP implementation. Two coauthors (GA and SC) facilitated these meetings, so we also used their ‘participant observations’ to identify HiAP- initiatives. Most of these data were posted on-line at the City’s HiAP web site.^15^ Next, we conducted 25 interviews with HiAP stakeholders between 2017 and 2019, that included nine City of Richmond staff representing seven different departments; ten CBO representatives that participated in the HiAP drafting and implementation processes, and; six Richmond residents that attended public meetings and live in the Iron Triangle neighborhood, one of the areas where HiAP-related projects are being implemented. Each interview lasted 30–45 min, and we asked interviewees to describe which HiAP initiatives they were aware of, how these projects were being implemented, and if they could describe any influences the HiAP-related projects were having on their organization, the local built environment and/or population health? None of the interviewees received compensation.

### Data Analysis

Interview transcripts were created from interviewee notes or recordings, and thematic analysis was used by each co-author to identify key practices and to interpret responses. The authors met several times to arrive at consensus on the common themes acknowledging that the authors’ subjective experience does play a role in the meaning making derived from the interview data.^16^

We also present survey data collected as part of the biannual Richmond Community Survey (RCS), which is administered by the National Research Center and the International City/County Management Association.^17^ This survey asks community members to rate their perceptions of quality of life, built environment, their economic and social well-being as well as city services. We report survey responses from 2007 to 2019 that included indicators previously identified in the HiAP Strategy as measures of health equity.^18^ Where possible we disaggregated survey responses by respondent race/ethnicity and neighborhood residence. Fourth, we describe data computed by the US Centers for Disease Control and Prevention, 500 Cities database, which provides estimated disease rates at the census tract scale for select health outcomes in the period before and after HiAP implementation.^19^

### What is Urban Health in All Policies?

As noted above, HiAP is an overarching framework that can be used for place-based governance that aims to shape the social and environmental determinants of health. HiAP, when used by city governments, often links disparate institutions and identifies opportunities for synergy across municipal practices that, when combined, might promote greater health equity. Examples of combinations of city planning practices that have the potential to promote greater health equity include land use and transportation, housing and economic development, environmental protection and racial justice and public safety and youth programs.^20^ Further, urban HiAP can also be collaborative with community stakeholders in ways that policy at larger scales, such as State and Federal government, may not. For example, local governments are mandated to include community participation in zoning, environmental impact assessment, and development reviews, providing an avenue for resident and CBO engagement with municipal decision making that can influence health.^21^ Urban HiAP has been shown to encourage local governments to make explicit how their policies and practice are or are not promoting health and where opportunities exist for greater population health benefits.^22^ Cities such as Boston, Baltimore, Seattle and a number of European, Latin American, and Asian cities have either endorsed or adopted HiAP.^23^ However, there is limited documentation on how these cities are implementing HiAP and whether or not they are tracking progress toward population health equity.

### The Implementation of Richmond’s Health in All Policies Ordinance

Richmond’s HiAP Ordinance was divided into six policy areas where implementing actions were defined.^18^ These six action areas included: (1) Governance and Leadership (2) Economic Development and Education (3) Residential and Built Environments (4) Full Service and Safe Communities (5) Environmental Health and Justice and (6) Quality and Accessible Health Care.

The Governance and Leadership action area was defined as the day-to-day management decisions within the City of Richmond, including the degree to which decisions are inclusive of different viewpoints, groups and cultures within the city, and reflect shared power. The HiAP Ordinance emphasized that both city and community leadership, collaborative decision-making and an attention to tackling institutional racism were all building blocks of a local health equity strategy. The Economic Development and Education action area emphasized that public policies should improve the financial status of residents, particularly wealth and relative levels of inequality, and opportunities for life-long learning. The HiAP Ordinance stated that addressing poverty, improving educational and employment opportunities for residents were all functions for the city and that these were key drivers of health equity. The Residential and Built Environment action area emphasized that housing quality and affordability, as well as that of community facilities, are drivers of population well-being. A Full Service and Safe Community was defined as one where public services were available, affordable, culturally appropriate, and of high quality, and that all neighborhoods were free of violence. The Environmental Health and Justice action area was defined as eliminating the burdens of pollution and improving access to healthy environmental “goods,” such as affordable, nutritious food, quality green spaces, clean energy, and other factors. Finally, the Quality and Accessible Health Care action area focused on ensuring that all residents had access to timely and affordable preventive care and disease treatment.^24^ What follows is our descriptive findings of the practices that emerged in each of the six action areas and then we present the indicators the HiAP Ordinance required the city to track in order to periodically measure progress toward health equity.

## Findings

### Governance and Leadership

Richmond’s HiAP is managed and led by the City Manager’s Office, which is the municipal department responsible for budgeting and coordinating all city departments. The HiAP Ordinance created the *Interdepartmental HiAP Implementation Team* and required that it have representatives from each city department. It was also charged with promoting more inclusive and transparent decision making around health equity goals, so community organizations and members were also invited to participate in the Interdepartmental Team’s decision making. According to one community member, the Interdepartmental Team is a forum for community members to ensure actions are serving every-changing population health needs, stating:

At first, we didn’t know really what HiAP was. The Interdepartmental Team meetings helped us identify ways we could partner with the city to enhance that we were doing around equity and inclusion. It also acted as a place where we could review proposed projects and insert health equity criteria. Through our participation with the city on the HiAP, we have collaborated on over $35M in successful grants from the State that are delivering real benefits to the Iron Triangle.

We also found a series of actions directly linked to the HiAP Interdepartmental Team that are aimed at improving health equity. First, the Interdepartmental Team developed a program that trains all city staff in the basics of the social determinants of health and health equity. Since the City of Richmond does not have a public health department a partnership with the county and academic institutions was created to implement these trainings on an annual basis. Second, the Interdepartmental Team reviews each city department’s 5-year budget plan and identifies indicators from the HiAP Strategy that can be linked to each department’s performance goals. For example, the Interdepartmental Team developed spatial equity indicators for the Engineering & Parks Departments, including measuring the extent to which street paving, lighting and sidewalk improvements served the poorest neighborhoods. The HiAP Interdepartmental Team also launched a new city program called the Governance Alliance for Race and Equity (GARE), which is part of a national network of cities working to advance racial equity through local policy making. Through this effort, the Interdepartmental Team has conducted trainings with all city employees about how to recognize and address inter-personal and institutional racial bias. The Interdepartmental Team drafted a new racial equity law in 2018, called *Resolution 93-18*, which mandated that every city department and council decision include a review of the impacts of decisions on racial and ethnic groups in the city and that all public meetings and documents be translated into the three most commonly used non-English languages in the city.^25^

The HiAP Interdepartmental team also worked with community-based organizations to create a web portal called *Transparent Richmond*. The Transparent Richmond platform includes easy to understand and access measures of governance, family and city economics, ratings of infrastructure and social services, measures of environmental quality, as well as community stories and resident narratives (https://www.transparentrichmond.org/). Transparent Richmond has centralized previously inaccessible public data, and according to a representative from the Richmond-based CBO called Urban Tilth:

Before HiAP was adopted we had a hard time getting regular public data. Once the portal was established, we were able to identify new groups that were food insecure and where they were living. We then partnered with a local clinic to launch a ‘food as medicine’ program called Veggie Rx. This project is delivering free produce to diabetic patients and enrolling them in a healthy eating and culturally relevant cooking class. With the city and county’s support through HiAP, we expanded our reach for this initiative.

### Economic Development & Education

The HiAP Interdepartmental Team helped coordinate a series of new programs focused on advancing access to education, employment and job training. The Interdepartmental Team coordinates a new program called the *Richmond Promise*, where the city offers college tuition assistance for all eligible Richmond graduating high school students and supports their success though on-going mentorship and network building. Since the Richmond Promise began in 2016, 2,918 Richmond students have received support, 68% of which were first generation college students and 72% were from low-income backgrounds.^26^

The Interdepartmental Team worked with the city’s Employment and Training division to bring a health and wellness component to a program called *RichmondBUILD*. *RichmondBUILD* offers formerly incarcerated and system-impacted youth training in construction skills and an apprenticeship program, where they learn basic carpentry, electrician and solar energy installation skills.^27^ According to Sal Vaca, former Director of Richmond’s Employment and Training Division, the HiAP Interdepartmental Team influenced their decision to create a ‘Healthy RichmondBUILD’ academy, stating: “Most of our students come from public housing or are formerly incarcerated. They didn’t get much exposure to healthy lifestyles. We saw they needed healthy food, maybe even counseling for anger and substance abuse issues, and to be in good shape. So, they [HiAP team] showed us that health didn’t have to be just health care. We now offer physical fitness, cooking classes, first aid/CPR certifications, and health screenings to our participants.” A mental health program was specifically set-up and connected trainees to counselors at a local community clinic. This aspect of the program recognized that, according to Vaca, “when our kids fight with each other it is often about unaddressed trauma and a cry for help, so we made sure they didn’t get kicked-out of the program and instead got supports we didn’t know were available for them.”

The Interdepartmental Team also worked with the City’s Department of Finance to create the Richmond Revolving Loan Fund. Through this fund, small businesses could apply for between $5,000–100,000 in funds for capital improvements, acquiring machinery or equipment, or for facade improvements. This program helped start the CoBiz project in Richmond, which is a new space adjacent to the city’s main train station, that supports the incubation of small enterprises by proving individualized business coaching, access to venture capitalists, and a physical ‘start-up’ space.^28^ The CoBiz site also includes workstations and an industrial kitchen where residents can create food stuffs for large-scale distribution. According to one community resident:

The Revolving Loan fund allowed me to expand my community garden and sell more at the farmer’s market. I took advantage of these programs to learn how to take this from a garden to a business that pays my rent and supports my family.

The Interdepartmental Team has also worked with the planning department to create new a new land use designation legalizing street vending and incentives for food-related economic development. According to HiAP Team member Gabino Arredondo, the zoning changes decriminalized food and other street vending in certain areas, which was a priority for many immigrant communities, The team also created a new zoning ‘overlay district’ for food production which lowered the regulatory and review thresholds for siting a food-related business while also offering tax incentives for hiring local people. The result was a food production cluster in a formerly vacant, industrial area of Richmond, and by 2017, several new businesses employing tens of residents, including Artisan Kitchen, Nutiva, Blue Apron, Galaxy Deserts, Urban Remedy & WholeFoods.^29^

### Full Service & Safe Communities

The HiAP Interdepartmental team recognized that at almost every public meeting, they heard that the top priority for residents was to improve public safety, particularly ending gun violence. In 2009, Richmond created a gun violence reduction program within the city called the Office of Neighborhood Safety (ONS). The Interdepartmental Team partnered with the ONS to improve partnerships across the city and connect the ONS to community-based organizations that could offer services to its participants. According to Richmond’s ONS Director, Sam Vaughn, the program:

[s]eeks those in the community that are not ‘at risk’ but those ‘creating the risk’ of gun violence. We don’t threaten them, but build trusting relationships with them, using street credible mentors. These mentors also offer cognitive behavioral therapy and other trauma-informed strategies, since we know many suffer from neglect and abuse. The City Manager’s office and staff have been instrumental in supporting this work and helping us connect those we engage from the streets to culturally responsive services.

**Figure 1.**
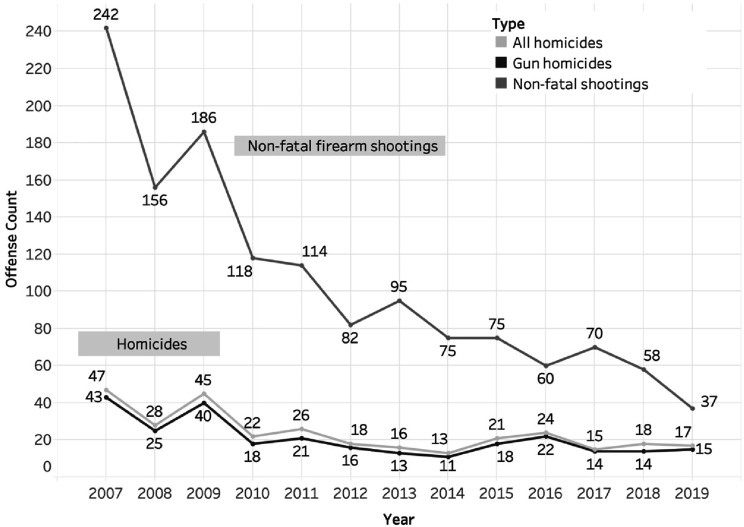
Richmond, CA, Gun Homicides and Shootings, 2007–2019.

A 2019 study of the ONS revealed that this program was responsible for a 55% reduction in gun homicides and assaults^30^ and these reductions have been sustained over time (Figure 1). According to Mike McLively of the Giffords Law Center to Prevent Gun Violence:

There is no other city in the US like Richmond that has had a comparable and sustained reduction in gun violence over such a long period of time. In our view, there is no other explanation for it except the consistent work of the ONS. Reducing gun violence then becomes the catalyst that brings economic development and other improvements to a community that has for too long been ignored, disinvested from and dehumanized by police.

In 2018, with the support of the HiAP Interdepartmental Team and pressure from residents, youth and community organizations, Richmond created a Department of Children and Youth. The Department created a youth advisory council that is responsible for allocating 3% of the City’s annual budget to programs, services and projects deemed important for improving well-being of Richmond’s young people.^31^ The HiAP Team helped lead a community needs assessment that identified funding priorities and community partners for the council. According to the Richmond Community Services Director, who is also the coordinator of the Department of Children and Youth:

We used a health equity lens from HiAP to identify the needs of young people in the community. Parents and youth participated in workshops that identified the on-going stressors in their lives. We asked them to identify the sources of those stressors for youth and what we as a city and in partnership with other service providers, could do to reduce those stressors. All this helped inform what the Department will focus on moving forward.

### Residential and Built Environments

The HiAP Interdepartmental Team took the lead in drafting Richmond’s *Fair Rent and Just Cause for Eviction Law* and established a Rent Control Program in 2017. The program was created to stop displacement of low-income residents and incentivize new affordable rental units. By 2019 there were 19,418 units within the rent control program. The HiAP team also created the Richmond Housing Renovation Program, which is a partnership between the city, the Richmond Community Foundation^32^ and Mechanics Bank. In this program, the city and CBO partners identify abandoned and blighted property, the foundation purchases these properties, the RichmondBUILD program renovates the homes, and the bank provides financing for low-income residents to purchase the newly renovated home.^33^ According to a Richmond resident:

Housing stress was the thing that we felt most threatened by. You know, being displaced and gentrification coming here like the rest of the Bay Area. The city’s housing programs really helped us get stabilized and own a home.

The HiAP Team also drafted and helped the city adopt an accessory dwelling unit (ADU), law, which allows property owners to increase the density on their sites and build more housing. According to Thao Nhi Phan, a resident:

We needed more affordable housing here and we didn’t trust the model where developers from somewhere else come in and build. This is just displacing people. The ADU rule allows local people to build housing that meets the needs of locals, like the many Asian immigrants that live with extended families.

A third key built-environment partnership created by the HiAP Interdepartmental Team was between the city and a community-based organization called Pogo Park. Pogo Park is a CBO created by residents of the Iron Triangle neighborhood, once Richmond’s most violent, polluted and impoverished areas. Through the creation of Pogo Park, residents came together to take back a neighborhood park, called Elm Playlot, from drug dealers and others.^34^ The group then worked with the city to apply for a State of California grant to revitalize the green space. The partnership has resulted in over $12M in funds from the State of California and private foundations that has not only helped rebuild Elm Playlot, but also to add a community center, and improve the street-scape in the Iron Triangle.^35^ The park has become the ‘heart beat’ of the Iron Triangle, creating jobs, safe play spaces, feeding hundreds of children every year from the community center, and bringing people together to heal in what was once Richmond’s most violent neighborhood.^36^

### Environmental Health & Justice

The HiAP Interdepartmental Team has created a series of partnerships with local organizations to achieve a range of environmental health objectives, such as reducing energy poverty and improving food security, and has co-led the drafting of Richmond’s Climate Change Action Plan. A strategy in the Climate Action Plan included a program for low-income households to receive free roof-top solar energy. The result is a partnership between the city and the non-profit organization called GRID Alternatives, which helps the City’s RichmondBUILD participants install rooftop solar. The funding for the program comes from the State of California’s Low-Income Weatherization Program. As of 2019, 1,718 new roof-top photovoltaic solar energy systems had been installed on low-income households at no cost, and a solar array, or farm, was created in Richmond to deliver free energy to 155 low-income renters that were not candidates for roof-top solar.

The HiAP Team also facilitated a partnership with the Bay Area Air Quality Management District to launch a “citizen science” community air monitoring program. This project provides real time data of air pollutants along the “fence-line” of the Chevron petrochemical refinery in Richmond.^37^ The HIAP Team helped connect UC Berkely researchers with the RYSE youth organization in Richmond, where young people helped set-up passive air samplers and conducted risk education for other young people.^38^ The community-based partnership also created a youth ‘air rangers’ program where students kept logs of pollutants. The data and mobilizing around air pollution are contributing to a community-led strategy for reducing air pollution from local industries.^39^

The HiAP Interdepartmental Team coordinates Richmond’s food policy council, which helps direct emergency food access to those in need and to plan for greater food security for all residents. Through the council, which includes community-based organizations and urban farmers, the city established a Seed Lending Library where residents can freely access seeds for their gardens and a fruit-tree give-away program. As mentioned above, one of the anchor food justice projects supported by the Interdepartmental Team is a “Food as Medicine” program which is delivering healthy, locally grown food to families in the Contra Costa Health Plan, a government run health care program.^40^ According to a leader within the Veggie Rx, food-as-medicine program:

What HiAP has helped the city do is find the thread that holds much of the community-based work together and know when to lead and when to step-back and support local organizations. Health equity can be a vague concept, but HiAP is helping us put it into action, and then tracking data to see how we are doing. We have a long way to go, but without a city framework, we would be fighting for more resources and recognition rather than implementing.

The Interdepartmental Team also coordinated a consortium of community-based projects into what is now called Richmond Rising, which won a State of California Transformative Climate Communities grant in 2022. In awarding the grant, the State explicitly recognized the city’s commitment to community partnerships, indicator tracking and linking place-based interventions to environmental and social justice.^41^

### Quality Health Care & Services

The HiAP Interdepartmental Team also recognizes that health equity demands that all residents have access to affordable, timely and high-quality care. A year after the HiAP Ordinance was passed, one of Richmond’s largest hospitals, called Doctor’s Medical Center, was closed.^42^ In responding to the ‘health care desert’ that was created by the hospital closure, the Interdepartmental Team worked with the State of California to increase enrollment in the state’s free medical care program, called Covered California. The city also partnered with the County to facilitate the construction of a new, full-service community health center in Richmond. The new clinic, operated by LifeLong Medical Care, includes a “Resilience Clinic” where University of California medical students diagnose and intervene on a patient’s adverse childhood experiences as part of the Collaborative Approach to building Resilience in Everyone (CARE) project^43,^^46^. This partnership highlighted that Richmond’s HiAP approach was committed to addressing the multiple determinants of health equity and is expanding the reach and quality of care for residents.

### Tracking Population & Place-Based Health Equity

**Figure 2.**
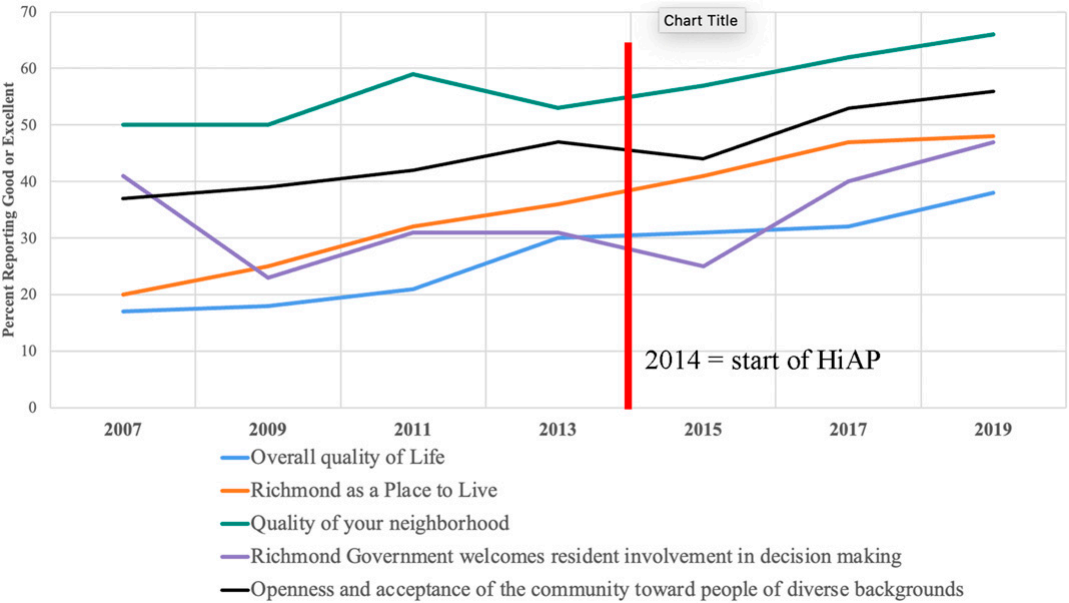
Richmond Community Survey, Health Equity Indicators, 2007–09.

As mentioned above, Richmond’s HiAP Ordinance included a set of indicators for tracking place and population-based drivers of health equity. A commitment was made by HiAP drafting stakeholders to primarily rely on publicly available data that was already being collected to populate the indicators, since they were concerned about the cost, availability, transparency, and longitudinal nature of the indicators.^44^ Five indicators were selected for tracking city-wide health equity using the Richmond Community Survey (RCS), and included resident ratings of: (1) The overall quality of life in Richmond (2) Richmond as a place to live (3) The quality of one’s neighborhood (4) the degree to which Richmond government welcomes resident involvement in decision making and (5) the openness and acceptance of the community toward people of diverse backgrounds. We plot the percentage of respondents rating each of these five indicators as “good” or “excellent” in the RCS from 2007 through 2019 in Figure 2.

The red line in Figure 2 indicates the time when the HiAP Ordinance was adopted in 2014. For all the indicators in Figure 2, the percent of respondents rating ‘good or excellent’ has increased between 2013 and 2019 by: 8 points (30–38%) for Overall Quality of Life; 12 points (36–48%) for Richmond as a Place to Live; 13 points (53–66%) for the Quality of Your Neighborhood; 16 points (31–47%) for ‘welcoming resident involvement in decision-making’ and; 9 points (47–56%) for the ‘Openness and acceptance of the community toward people of diverse backgrounds.’

**Figure 3.**
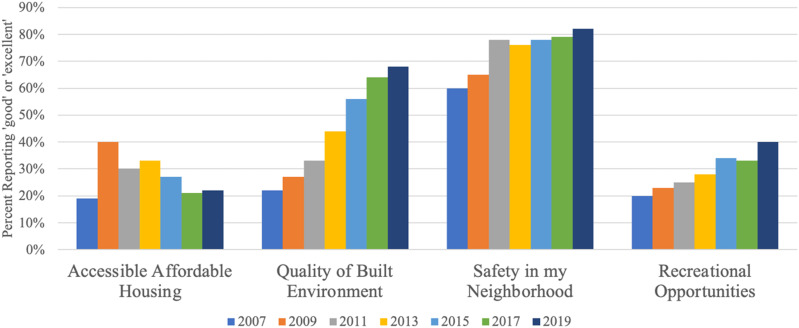
Richmond Community Survey, % Reporting Good or Excellent, 2007–2009.

Additional city-wide indicators from the RCS were selected within each HiAP Action Area. We report on four of those indicators, including accessible affordable housing, the quality of the built environment, safety and recreation opportunities (Figure 3). Like the perceptions of equity reported in Figure 2, these indicators except for affordable housing, are trending positively, but none of the changes are statistically significant.

**Figure 4.**
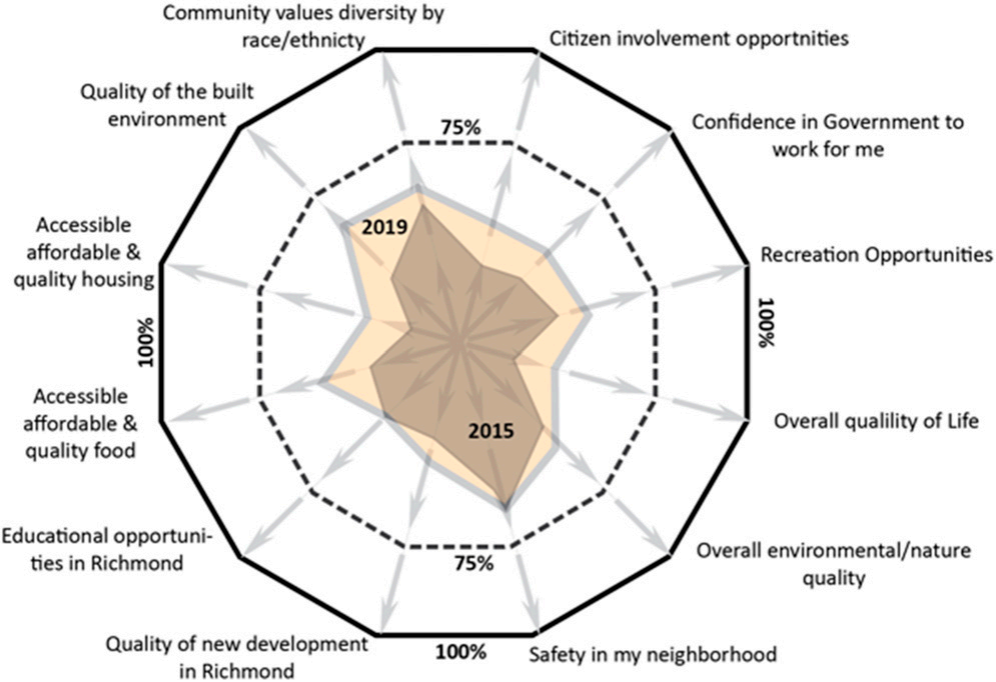
Percent of Richmond, CA, Iron Triangle residents responding good or excellent in the Richmond Community Survey, 2015 and 2019.

As noted, the Iron Triangle neighborhood was chronically the unhealthiest in Richmond, and we present survey responses from Iron Triangle residents to illustrate how Richmond’s HiAP is tracking place-based indicators of equity. The ‘health equity polygon’ presented in Figure 4, was created during the HiAP drafting process to display and track a set of related indicators, and to communicate a more holistic portrait of progress toward health equity in each neighborhood. The twelve indicators all come from the RCS and included equity measures of decision-making processes (i.e., citizen involvement in decisions and racial/ethnic inclusion), overall quality of life, and access to quality housing, recreation, education, built environments and healthy foods (Figure 4). For almost all health equity indicators, respondents in 2019 from the Iron Triangle neighborhood reported improvements, although the goal of 75% reporting positively (dashed line) was not achieved (Figure 4).

**Figure 5.**
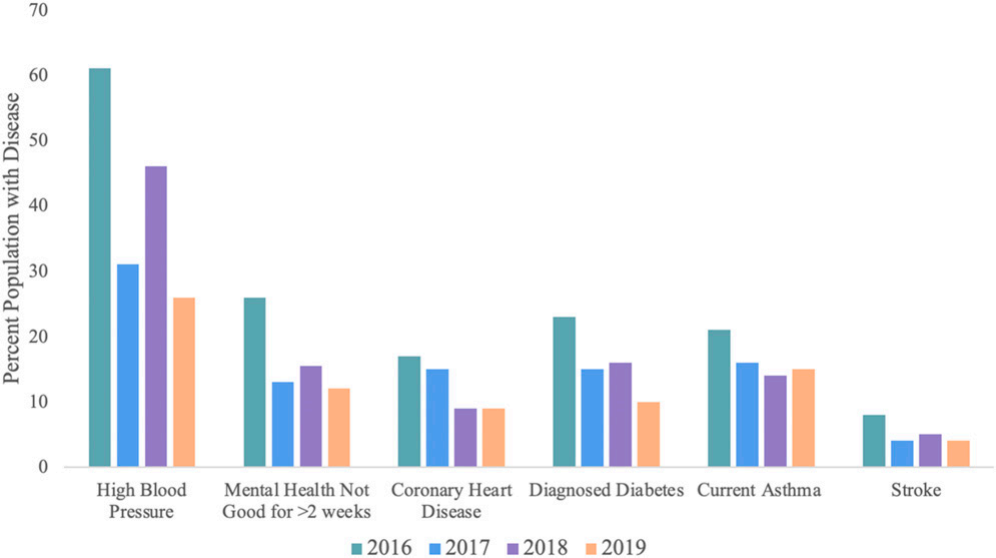
Richmond, California, Iron Triangle Neighborhood Health Data from the CDC.

Finally, the HiAP Ordinance uses disease data reported by the US, Centers for Disease Control and Prevention, to track population health progress. Here, we present the CDC generated disease rates only for the Iron Triangle neighborhood, including the percent of residents with high blood pressure, mental illness, diabetes, asthma, heart disease and stroke. Like the perceptions data presented above, the disease outcomes seem to be trending downward between 2016 and 2019, except for asthma (Figure 5).

### Pursuing Health Equity in Local Government

Local HiAP implementation can only achieve health equity when community organizations and residents are leaders and beneficiaries. We found that the implementation in Richmond has an explicit community engagement strategy, but more work can be done to center resident voices and expand their decision-making power. For example, an activist with the Laotian Organizing Project, a CBO in Richmond, commented on their perception of the HiAP implementation process: “We feel like city staff are partners with our community after years of being ignored or outright hostile. Asian people know this is a safe place for them and the city has made that clear through holding Chevron accountable, ensuring all meetings are available in our languages, and staff having cultural humility and competencies.” Former Richmond Chief of Police, Chris Magnus, stated:

The HiAP process allowed us to make a public health argument for safety and putting resources into things like stopping human trafficking. It also showed us that we could achieve more by working in partnership with other groups preventing violence and that improving parks and street lighting, maintaining vacant lots and housing, and getting people back to work, can have an impact on safety and well-being.

One San Francisco Bay Area newspaper reported that the changes in Richmond were nothing short of a ‘renaissance,’ stating:

A new spirit in city government has helped transform industry, the quality of life in the city, and Richmond’s grim reputation. The city has undergone a facelift, citizens are attending community meetings and events in unprecedented numbers, and new businesses — many of them green — are bringing economic opportunities back to town.

While other cities are desperately contending with debilitating budget deficits and struggling to maintain public safety and other basic services, Richmond has produced balanced budgets and enjoys a full complement of police officers. The combined efforts of city departments and community members have resulted in meaningful reductions in violent crime. And the city has completed numerous civic and neighborhood revitalization projects that have given Richmond a new air of vitality and community health.^45^

## Discussion

This case study has highlighted the nuanced practices that fall under the umbrella of an urban HiAP Ordinance. One key finding is that Richmond’s HiAP Ordinance has institutionalized a new equity-promoting governance structure (i.e., the HiAP Interdepartmental Team). However, it is unclear whether community voices are driving the Interdepartmental Team’s decision-making or how much power CBOs have in this forum. What is clear is that absent the Interdepartmental Team, many programs, practices, partnerships and policies would not be attentive to the community’s definition of health equity enshrined in the HiAP Ordinance.

Our descriptive case study of Richmond’s experience with HiAP is consistent with research exploring the challenges and opportunities of implementing HiAP in local government. For example, Molnar et al. 2016, found that in reviewing 16 HiAP initiatives, implementation was facilitated by integrating health into other policy agendas (i.e., sustainability), the use of scientific evidence to demonstrate its effectiveness and using health impact assessment to make policy coordination for public health outcomes more feasible.^46^ The importance of institutionalized health equity practice and governance, and how HiAP can accomplish this, has been recognized in other evaluations. In a review of multiple HiAP strategies at the municipal scale, Guglielmin et al (2018) found that the keys to successfully addressing health equity included developing cross-sector relationships within government, incorporating health equity into everyday decision making processes, expanding workforce capacity, linking HiAP to new sources of funding, and ensuring there is on-going evaluation reporting about impacts and challenges.^47^ Another case study of municipal implementation of HiAP in Kuopio, Finland, found that the keys to success were having a common goal, leadership from policy and political elites, and the presence of committed staff charged with facilitating HiAP implementation.^48^ This study did not explore nor address whether or not a measure of ‘success’ included civil society participation or whether or not the implementing strategies addressed existing, local drivers of health inequities. However, a scoping review of municipal implementation of HiAP by Lilly at al., found that factors of implementation success included having a clear definition of health (equity), using local evidence to highlight existing inequities, the presence of policy champions or entrepreneurs, and ensuring there was a dedicated institutional structure and funding stream for HiAP implementation and monitoring.^49^ Finally, Amri (2022) argues that municipal government leadership in HiAP is essential for achieving more healthy and equitable cities.^50^

Richmond’s HiAP implementation strategy may also offer insights for other local governments and CBOs on how to affordably measure progress toward health equity.^51^ Richmond’s use of existing survey data is helping the city avoid expensive, time-consuming data collection while offering easy to understand, publicly accessible, longitudinally collected indicators. We found few studies of local HiAP that offered community-gathered data, tracked these for different population groups and neighborhoods, or made these data easily accessible to public stakeholders.^47,52^ A key limit is that our data collection was focused only through 2019, so the challenges and equity impacts of COVID-19 are not captured. Surely the COVID-19 pandemic raised new community health equity challenges for future HiAP implementation.

## Conclusions

Health in all Policies offers the potential for local governments to lead on health equity with, not just for, community partners. This case study has suggested that HiAP at the local level can act as a framework for policy and practice and may best promote health equity through the creation of meaningful partnerships with community stakeholders. Clearly more work is needed to determine if and how health equity is being achieved in Richmond, CA, and what role HiAP might be playing in these processes. This case study has offered preliminary findings and descriptive data in the hope of stimulating further dialogue and study on the potential and promise of municipal HiAP for promoting greater health equity.
